# How Does Gamification Improve Purchase Intention? Through the Lens of Perceived Brand Coolness and Time Poverty

**DOI:** 10.3390/bs14121226

**Published:** 2024-12-19

**Authors:** Yingchuan Liao, Fei Zhou, Youcheng Chen, Yenchun Jim Wu

**Affiliations:** 1 College of Business Administration, Huaqiao University, Quanzhou 362021, China; liaoyc@fafu.edu.cn (Y.L.); feiz@hqu.edu.cn (F.Z.); 2 College of Digital Economy, Fujian Agriculture and Forestry University, Fuzhou 350001, China; uceng@fafu.edu.cn; 3 Graduate Institute of Global Business and Strategy, National Taiwan Normal University, Taipei 10645, Taiwan; 4 MBA Program in Southeast Asia, National Taipei University of Education, Taipei 10671, Taiwan

**Keywords:** gamification marketing, purchase intention, perceived brand coolness, time poverty, consumer behavior

## Abstract

Gamification has been extensively employed in marketing practices to meet the diverse needs of consumers. Previous research suggests that gamification marketing plays a pivotal role in influencing customer purchase intention. However, the precise mechanism through which gamification marketing impacts purchase intention requires further investigation. Drawing on the self-determination theory (SDT), this study explores the relationship between gamification marketing and purchase intention, with customers’ perceived brand coolness as a mediating variable and time poverty as a moderating variable. Using data collected from 184 participants in the experiment, our research demonstrates that, in comparison to non-gamification marketing, gamification marketing significantly influences purchase intention. Furthermore, perceived brand coolness emerges as a mediating factor in this relationship, providing new insights into the gamification mechanism. Customers who are in low time poverty exert more perceived brand coolness and purchase intentions compared with high time poverty in the context of gamification marketing. This study expands the research of gamification by introducing perceived brand coolness to the relationship between gamification marketing and purchase intention. It also contributes to the study of time poverty under the context of gamification marketing.

## 1. Introduction

One would be attracted by activities that are funny and enjoyable which can be initiated by utilizing game design elements. Gamification refers to the integration of game elements into non-game situations [1,2]. Integrating game mechanisms with personal disposition results in fun and enjoyment [3,4]. For example, Starbucks uses reward cards as gamification to create consumer engagement with the brand [5]. Alibaba integrated “11·11” Tmall shopping carnival activities with interactive games. Customers received lucky money as a reward by playing games on the Tmall shopping platform by completing tasks such as browsing or inviting friends. This lucky money can be used for payments during the “11·11” sales promotion. Pinduoduo has introduced the Duoduo Orchard Game Land on its platform, where users receive a box of mature fruit in return for planting trees on the online website and completing tasks like browsing, placing orders, sharing, or inviting friends. Additionally, consumers earn points that can be redeemed for products. When a participant’s operational ability aligns with the game difficulty, they develop a psychological state that effectively enhances customers’ purchase intention [2,6]. The above information shows that gamification marketing is an effective way to influence practitioners on branding strategies.

However, there is currently a lack of empirical studies discussing the effectiveness of gamification in brand attitude. Consumers may choose a product because they perceive it as cool or because others in a given social context regard it as “cool” [7,8]. Consumers derive enjoyment from the perception of being “better” through purchasing cool products [9,10]. Perceived brand coolness mediates the relationship between gamification and brand awareness, as well as brand value [11,12]. Similarly, attitude serves as a mediator in the relationship between gamified advertisements and purchase intentions [13]. According to self-determination theory (SDT), when individuals feel greater motivation from activities, they tend to demonstrate higher levels of engagement [14,15]. A positive gamified experience can attract customers and positively impact their attitudes and behaviors which contribute to an immersive experience known as “flow experience”, making people feel a sense of coolness. The phenomenon of coolness in a branding context can significantly motivate consumer responses [16].

As modern life accelerates, consumers facing high time poverty struggle to allocate sufficient time for important activities, leading to difficult trade-offs [17]. The literature indicates that individuals experiencing time poverty may face cognitive behavior blocks and accumulate negative emotions, leading to short-term decision making and unconscious disregard of existing information, thereby impacting intertemporal decisions [18,19]. When gameplay is mandatory or occurs under time poverty, gamified interactions may not necessarily facilitate positive brand connection outcomes [20]. When consumers are in high time poverty, they may not intrinsically be motivated by gamification activities for they are tired of dealing with multifarious works [21]. Consumer characteristics and usage context characteristics as moderating variables have been discussed previously [22,23]. We consider time poverty as a moderating variable in the relationship between gamification marketing and purchase intention for time poverty commonly exists with the rapid pace of life.

Based on self-determination theory (SDT), this paper aims to explore how gamification marketing influences users’ purchase intention with the mediating effect of perceived brand coolness and the moderating effect of time poverty. Studies about the impact of gamified marketing on consumer behavior were mostly conducted from the perspective of fun and flow experience. While there are few empirical studies from the perspective of perceived brand coolness. In addition, the boundary conditions of gamified marketing were discussed with a focus on individual characteristics or game types. This paper introduced time poverty as a moderating effect which would be a supplement to the existing research. The research formulates hypotheses and adopts a 2 × 2 experiment to verify the relationships. In the subsequent sections, we review prior literature on gamification marketing, then establish hypotheses and a research model describing how gamification marketing affects customers’ purchase intentions. Data were collected mostly from young individuals in China and analyzed through independent sample T-test and regression analysis. Finally, we concluded this work with practical implications, limitations, and directions for future research.

## 2. Theoretical Background and Research Hypotheses

### 2.1. Self-Determination Theory (SDT)

Self-determination theory (SDT) frames a motivation model for understanding human behavior. People can be motivated when their needs for competence, autonomy, and relatedness are fulfilled [24]. The motivations consist of intrinsic and extrinsic ones. Extrinsic motivations refer to rewards externally imposed, whereas intrinsic motivations refer to the above three basic psychological needs [25]. Self-determination theory (SDT) explains that external interventions can be transformed into intrinsic motivations [26]. Considering that intrinsic motivation is an important way to enhance users’ voluntary participation behavior, some scholars have pointed out that the application of game elements can meet these three basic needs of people, increasing intrinsic motivation, and enhancing their voluntary participation [27,28,29]. The use of gamified systems increases engagement, and this behavioral change was attributed to the influence of intrinsic motivation [30]. Gamification is often supposed to be an effective instrument to foster motivation which can be immense during playing.

### 2.2. Gamification and Purchase Intention

Researchers define gamification as the utilization of game design elements in non-game contexts to enhance user enjoyment and engagement. It was specifically described as “the application of game-design principles to change behaviors in non-game situations” [1,31]. The mechanics of a gaming system are derived from a set of tools, including points, levels, leaderboards, badges, and challenges [32]. Gamification is characterized by challenging tasks and interactivity [33,34]. Managers and researchers have advocated for the use of gamification in platform design due to its interactive capabilities [31,35]. Gamification has been suggested as an effective tool for motivating people to increase physical activity and improve mood [33]. Werbach and Hunter [36] considered the motivation mechanism as the essence of gamification with the core of providing fun and increasing users’ voluntary participation.

Positive experiences and a sense of enjoyment caused by gamification may enhance marketing effectiveness, influencing engagement, attitude, purchase, repurchase, and retention [37,38]. Gamification has proven to be useful for changing behavior [39,40]. Incorporating game elements into marketing activities has been proven effective in increasing consumer engagement [5]. Firms design gamification strategies with the aim of enhancing customer satisfaction, loyalty, and engagement [41]. Several studies have demonstrated that gamification influences purchase intention [6,42]. Games with progressive difficulty, beautiful interactive interfaces, and coordination between game design style and the product are well appreciated to maximize the purchase intention [43]. Müller-Stewens et al. [44] assume that gamification entertains customers mainly in an effort to attract them to buy [45]. Retailers leverage game mechanics in online shopping to transform it into an entertaining activity, with a focus on improving consumers’ attitudes and overall experiences [46]. Under the condition of challenge and competition, participants would show more enthusiasm for the activity with positive emotions, potentially improving participants’ good impression of products [47]. Harwood and Garry [35] found through empirical investigation that a gamified experience environment generate and reinforces positive relational outcomes, including customer loyalty and relationship development. Psychological states evoked by the motivational affordances of gamification activities foster customer engagement, ultimately leading to purchase outcomes [48,49]. Therefore, previous studies examining the relationship between games and customer buying behavior provide a solid theoretical foundation for this research hypothesis. Based on this, we propose the following:

**H1:**  *Compared with non-gamification marketing, gamification marketing promotes higher purchase intention among customers.*

### 2.3. Mediating Effect of Perceived Brand Coolness

Coolness is the “most precious natural resource” which is capable of transforming an otherwise substitutable product into something “fantastically valuable” [50]. Warren et al. [51] used a grounded theory approach to identify characteristics of cool brands that are popular, high status, subcultural, appealing, and so on. Bagozzi et al. [52] proposed five dimensions related to brand coolness: personal cool, originality, usability, high status, and reliability. In the context of creative tourism, perceived coolness with uniqueness, usefulness, attractiveness, and subculture level positively affects satisfaction and place attachment [53]. Warren et al. [54] found that coolness is rooted not only in desirability but also in autonomy, highlighting the dynamic nature of coolness as societal adoption of such behaviors evolves.

Studies have consistently shown that embedding coolness in brands and products can positively impact consumer attitudes and behaviors [55,56], leading to significant marketing value [16]. Loureiro et al. [57] posited that consumers’ passionate desire for brands was an outcome of brand coolness. Brand coolness leads to various outcomes, such as perceived value [56], attachment and loyalty [58], intention to use, satisfaction [59], attitude, and increased spending [51]. Research by Trank et al. [8] showed that perceived brand coolness exerts positive impacts on brand relationship outcomes (i.e., brand satisfaction, brand love, and brand advocacy). Saavedra Torres et al. [60] obtained a result that brand coolness mediates the effect of brand-to-brand communications on brand sincerity.

Under the context of gamification, the act of participating in games can have a favorable impact on perceived brand value, fostering a strong emotional connection between customers and brands [61]. Arya [62] examines the mediating role of consumers’ brand engagement on the relationship between gamification marketing activities and consumer-based brand equity. Consumers’ perceived brand coolness has a positive effect on purchasing and using behavior [63] through hedonic value and functional value of the brand [64]. From the perspective of cool types, gamification marketing is particularly appealing and interesting. On the one hand, game activities have naturally been participated mostly by individuals who are intrinsically motivated personally by hedonic values. On the other hand, gamification marketing activities are characterized by funny and interesting flow experience which is consistent with brand coolness. Previous literature considered perceived funny or flow experience as an explanation mechanism for the effect of gamification on consumer behavior [65]. However, empirical research from the perspective of perceived brand coolness is inadequate. Building on the above discussed, this paper posits that gamification strengthens the brand experience and transfers the positive perception of the brand to consumer purchase behavior. In this sense, we propose the following Hypothesis 2.

**H2:**  *Perceived brand coolness mediates the relationship between gamification marketing and purchase intention.*

### 2.4. Moderating Effect of Time Poverty

Time poverty was possibly first introduced by Vickery [66] in the US context, aiming to identify households with insufficient available time to maintain a non-poverty standard of living. Urakawa et al. [67] defined time poverty as the situation where individuals lack sufficient time for rest and leisure after accounting for work time. Researchers have categorized time into work time and leisure time, necessary time, and discretionary time. Discretionary time refers to the time available for personal care [68]. Individuals are considered to be in a state of time poverty when necessary time exceeds a certain threshold or discretionary time falls critically low [69,70]. Studies have argued that time poverty increases decision-making difficulties [71,72]. Recent scientific evidence suggests that feeling time poverty can negatively affect subjective well-being, including life satisfaction, positive affect, mental health, and work performance [69,73]. Time poverty may have both positive and detrimental effects on performance, depending on the degree of time poverty and subjective appraisals of the relationship between perceived demands and available resources. This can lead to poor decisions that worsen one’s state of deprivation [18].

Decision makers can search for all possible or optimized strategies to solve problems when they are under sufficient time conditions. However, when the time allocated to decision makers is less than what is actually needed or perceived, it may cause emotional experiences of time pressure, affecting the decision process. Consumers inevitably reduce their purchase intention when they are unsure if a product can meet their needs under high time poverty conditions [74]. When consumers experience time poverty to complete the game, their cognitive elaboration about the brand will be diminished [20] which would cause poor perceived brand coolness.

Although gamified marketing can effectively enhance customer value and encourage customer engagement, the marketing effect varies with factors such as age, income, education, and experience [22,38]. Prior literature has proposed age, gender, income, ability, education, and product characteristics as boundary conditions of the gamification effect. Individual participation motivation (express, compete, explore, collaborate) moderates the relationship between gamification and consumer engagement [75]. Gamification can either enhance engagement or detract from it, depending on its implementation. For instance, when firms impose time restrictions during gameplay, consumers may feel disengaged from the brand, resulting in weakened self-brand connections due to the perceived lack of adequate time to play [20]. Personal characteristics and brand product characteristics as boundaries have been discussed in previous studies [48,49]. However, the condition of playing games has referred less, especially in existing empirical research. We expect time poverty to have different effects on perceived brand coolness as well as purchase intention.

Therefore, it is reasonable to assume that consumers with low time poverty are more likely to have a higher purchase intention under the condition of gamification marketing. In addition, customers would perceive more brand coolness when they are in low time poverty because they are in a rest and leisure condition. The following has been hypothesized:

**H3:**  *Time poverty has a moderating effect on the relationship between gamification marketing and purchase intention.*

**H4:**  *Time poverty has a moderating effect on the relationship between gamification marketing and perceived brand coolness.*

### 2.5. Theoretical Framework

Self-determination theory (SDT) underscores the importance of intrinsic motivation in driving self-determined behaviors [76]. Intrinsic motivation can be fostered through participation in challenging tasks or enjoyable activities [24], making it a critical consideration in gamification strategies designed to enhance consumer engagement. Some notable works argue that intrinsic motivation can be achieved through gamification [1,77]. Gamification serves as both intrinsic and extrinsic interventions through various game elements that support competence and autonomy [25]. Game elements could be incorporated within non-gaming products and services to provide enjoyment and potentially increase consumer retention [60]. An empirical study conducted by Bitrián et al. [78] concluded that game elements impact the satisfaction of basic psychological needs (i.e., competence, autonomy, and relatedness) of users, facilitating autonomous motivation. Gamification elements fulfill consumers’ psychological needs by providing rewards, fostering competition, and promoting autonomy [77,79]. Features such as badges, rewards or points serve as extrinsic motivators, offering a framework to explain individual behaviors in game-based activities [6,30]. For instance, in the online Duoduo Orchard Game Land, individuals were motivated by online planting games through which they received enjoyment and fun.

Gamified interactions are considered a main form of consumer entertainment with the objective of brand building, and providing consumers with positive experiences [20]. Integrating brand elements with game stories stimulates a new emotional relationship between the consumer and the brand, increasing customers’ positive attitude towards the brand. Moreover, brands can be promoted more efficiently with games, targeting a broader audience [80,81]. However, not all gamification marketing activities yield the expected direct effects. In an experiment using apps on smartphones in a brick-and-mortar store, Högberg et al. [30] found that gamification does not impact the choice of a target product but only has an influence when users become genuinely engaged with the application. When one can receive funny experiences from the brand during their buying process, we may perceive it as a cool thing which causes a buying behavior. Customers would receive a flow to the brand offering coolness. Under the low time poverty condition, it can be supposed that the influences are strengthened so customers can receive enough time to enjoy those brand coolness perception experiences.

**Figure 1:**
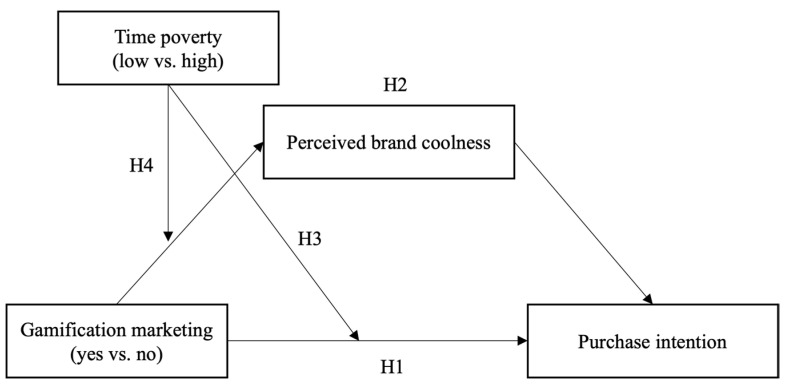
Research Model.

Building on the discussion above, this study proposes perceived brand coolness as a mediator and time poverty as a moderating variable in the relationship between gamification marketing and consumer purchase intention. The research model is depicted in Figure 1.

## 3. Materials and Methods

The research employed lipstick as a stimulus to compare the impact of gamification and non-gamification marketing on purchase intention. Additionally, it examined the mediating effect of perceived brand coolness and the moderating effect of time poverty.

### 3.1. Method

The study adopted experimental method to examine the effects of gamification on purchase intention, employing a 2 (gamification marketing: yes vs. no) × 2 (time poverty: high vs. low) between-participant design.

### 3.2. Experiment Design Procedure

Given that most customers for gamified marketing and cool brand products are young, participants were selected more from the young category and randomly assigned to one of the experiment conditions. The experiment included the following components: introduction, personal information, screening items (emotional condition, knowing, and liking extent), TSST (Trier Social Stress Test) of time poverty, gamification and non-gamification marketing perception measurement scale, perceived brand coolness measurement scale, time poverty perception measurement scale, purchase intention measurement scale, price item and emotional state items (to ensure participants’ engagement), gender, age, and monthly income. Measurement items of time poverty, and perceived brand coolness used a 7-point Likert scale. The lipstick brand used a virtual brand name in order to eliminate the influence of brand familiarity. In the gamification conditions, participants played a lipstick turntable game, with the incentive of earning points and securing a position on the leaderboard. The top 100 players received a one-yuan discount reward and could view the overall friends’ leaderboard. The game could be played multiple times, but only the best result was recorded. Participants could socially share the game through a website with a QR code, enhancing the promotion effect. In the non-gamification conditions, participants were informed about a traditional online promotion activity where the first 100 customers would receive a one-yuan reward only.

### 3.3. Time Poverty Manipulation

The Trier Social Stress Test (TSST) developed by KirSchbaum [82] is used for time poverty control. Participants were told they were attending an important job interview, requiring them to complete an oral calculation test within a limited time without using tools. High time poverty and low time poverty groups received different calculation tasks. High time poverty group was assigned the task of reducing a number from 2063 to 18 in 60 s, allowing for six iterations. In contrast, the low time poverty control group was given the task of calculating 1 plus 5 in 30 s, allowing for four iterations. Following the task, participants answered questions regarding their perceived time pressure during the calculation and how often they felt rushed or pressed in the past period, aiming to validate the manipulation of time poverty. This rigorous time poverty manipulation procedure ensured that participants’ perceptions of time poverty were effectively manipulated in the experiment.

### 3.4. Measurement of Experimental Variables and Manipulation Checks

Measurement items referenced from Chen and Chou [58] were used to measure perceived brand coolness. It could be listed as the followings, “Participating in advertising just now makes me look cool”, “When I think of ADs that are cool, advertising just now comes to mind”, “When I visit advertising just now, my response often is something like ‘That’s cool!”, “Advertising just now has some cool features”, and “If I made a list of cool advertising, advertising just now would be on it”. Measurement of purchase intention referenced from Tezer and Bodur [83] including 3 items (“If necessary, I will consider buying this brand product”, “I intend to purchase this product in the near future”, “I would like to recommend this brand to others”). The measurement scales were adapted from past research studies and employed a 7-point Likert scale ranging from 1 (strongly disagree) to 7 (very strongly agree).

In addition, two extra manipulation checks were conducted: gamification marketing check and time poverty manipulation check. For gamification/non-gamification marketing manipulation check, a 7-point Likert scale ranging from 1 (not at all likely) to 7 (very likely) was used to measure the gamification/non-gamification perception. Participants responded the item “The advertisement can achieve marketing effect”. Measurement items referenced from Roxburgh [84], “I feel being pressed for time during the calculation” and “In the past period of time, I felt rushed and pressed for time” (1 = Rarely, 7 = Very often) were used to check time poverty experimental manipulation.

## 4. Results

A total of 205 participants performed the experiment. Participants who finished the experiment incompletely or unqualifiedly were excluded. Finally, 184 participants effectively completed the experiment. The descriptive statistics are shown in Table 1. As noted in Table 1, our target respondents mainly comprise young people, which is also the major group of game players [48]. We collected the age of consumers, so we could compare the effect of the two different age groups between 27 and 45 and 19 and 24. The results show that both of the groups are balanced in the gamification effect on purchase intention. The gender balance (21.2% male to 78.8% female) is also in line with the real-life lipstick market situation (26.8% male to 73.2% female) reported by “Survey Data of Lip Makeup Development and Consumption in China”. Overall, our sample is representative of the target population.

### 4.1. Reliability and Validity

We assessed the reliability and validity of variables by conducting Cronbach’s alpha (CA) value and KMO value. Cronbach’s alpha (CA) values were as follows: perceived brand coolness (0.867), perceived time poverty (0.715), and purchase intention (0.853). The reliability values were all greater than the recommender threshold of 0.7, indicating that the measurement model was reliable. And the KMO value was 0.827 which was greater than 0.7 and considered adequate.

### 4.2. Manipulation Checks of Experiment

Gamification marketing was used as a grouping variable (1 = gamification marketing, 0 = non-gamification marketing) and consumers’ subjective gamification perception was used as a test variable to conduct an independent sample *t*-test. It can be found that the mean value of the gamification group was higher than non-gamification (M_gamification marketing_ = 4.98, SD = 1.32; M_non-gamification marketing_ = 3.49, SD = 1.21, *t* = 3.03, *p* = 0.003). So, it can be concluded that the gamification manipulation experiment was successful.

Time poverty was used as a grouping variable (1 = high time poverty, 0 = low time poverty) and consumers’ subjective time poverty perception was used as a test variable to conduct an independent sample *t*-test. It can be concluded that the mean value of the high time poverty group and low time poverty group have a significant difference (M_high time poverty_ = 4.35, SD = 1.54; M_low time poverty_ = 2.31, SD = 1.51, *t* = 2.54, *p* = 0.012). Hence, the time poverty manipulation experiment was successful.

To sum up, both gamification marketing and time poverty manipulation check receive a good result which could better detect the subjective perception of participants.

**Figure 2:**
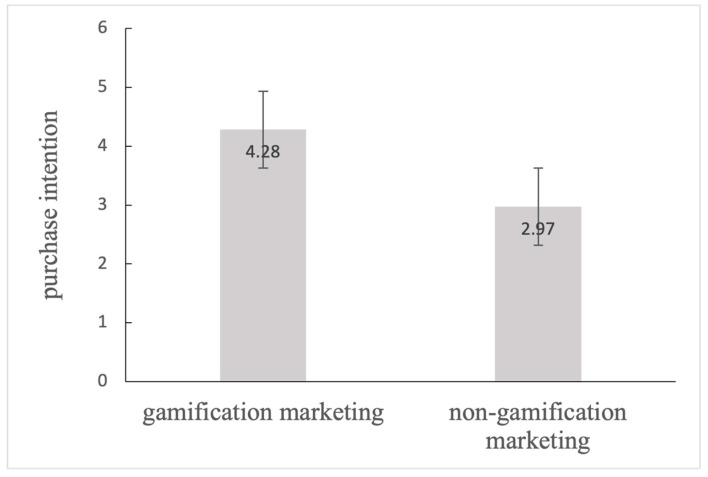
Purchase Intention of Gamification Marketing and Non-gamification Marketing.

Gamification marketing was used as a grouping variable (1 = gamification marketing, 0 = non-gamification marketing) and consumer purchase intention was used as a test variable to conduct an independent sample *t*-test. Figure 2 shows that the mean value of the gamification group was higher than the non-gamification group (M_gamification marketing_ = 4.28, SD = 1.14; M_non-gamification marketing_ =2.97, SD = 1.26, t = −3.565, *p* = 0.009). Hence, gamification marketing facilitates purchase intentions much more than non-gamification marketing.

### 4.3. Regression Analysis

**Figure 3:**
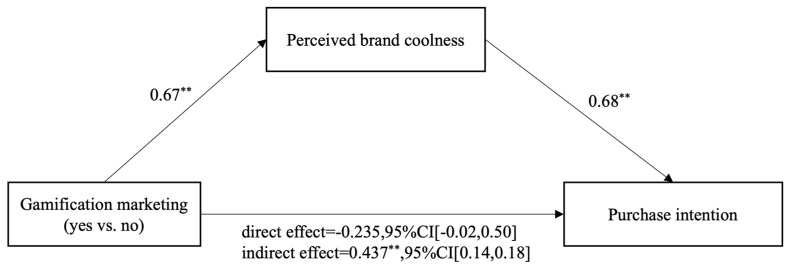
The Mediating Effect of Perceived Brand Coolness. Note. ** means *p* < 0.01; CI = confidence interval of the difference.

In order to further verify the mediating effect of perceived brand coolness, we ran a Bootstrap mediation analysis by using model 4 in the Process Macro with perceived brand coolness as the mediating variable and purchase intension as the dependent variable. The results are shown in Table 2 and Figure 3.

To sum up, gamification marketing has a significant positive effect on purchase intention (B = 0.64, *t* = 3.43, *p* < 0.01) and a significant positive effect on perceived brand coolness (B = 0.67, *t* = 3.78, *p* < 0.01). Perceived brand coolness has a significant positive effect on purchase intention (B = 0.68, *t* = 13.66, *p* < 0.01). Meanwhile, the lower interval and upper interval of the bootstrap 95% confidence interval of the indirect effect of gamification marketing on purchase intention do not contain zero which indicate that the indirect effect of gamification marketing on purchase intention through perceived brand coolness is significant. So, Hypothesis 2 is supported and shown by Figure 3.

Model 8 of the Process Macro was used to examine the moderating effect of time poverty. The interaction item is gamification × time poverty, which is set as an independent variable. It can be concluded that the moderating effect is significant. Therefore, perceived brand coolness mediates the relationship between gamification marketing and purchase intention.

**Figure 4:**
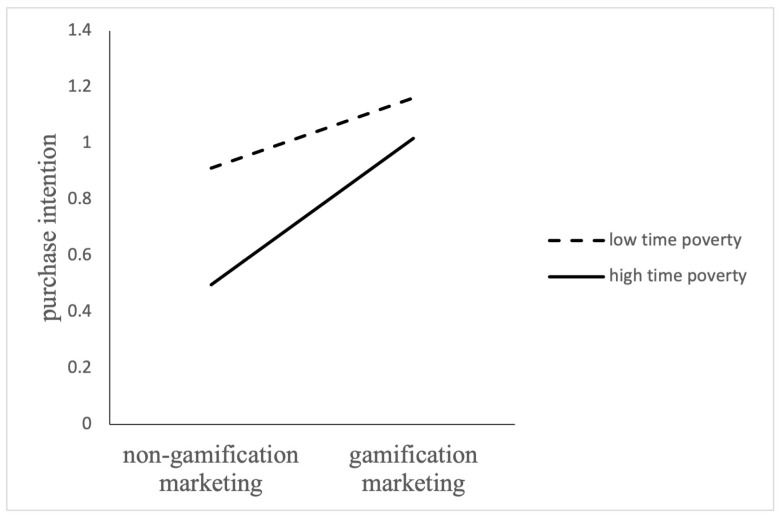
The Moderating Effect of Time Poverty on the Relationship Between Gamification Marketing and Purchase Intension.

Furthermore, according to Figure 4, gamification marketing has a significant positive impact on purchase intention for participants with low time poverty perception (M − 1SD) (B = 0.76, *t* = 3.02, *p* < 0.001) and with normal poverty perception (M) (B = 0.56, *t* = 1.76, *p* < 0.001). However, for participants with high time poverty perception (M + 1SD), gamification marketing has no significant effect on purchase intention (*p* = 0.99 > 0.001). The results show that with the rise of perceived time poverty, the positive effect of gamification marketing on purchase intention decreases gradually. Under high time poverty conditions, both gamification and non-gamification marketing have no significant effect on purchase intention. So, Hypothesis 3 is supported.

**Figure 5:**
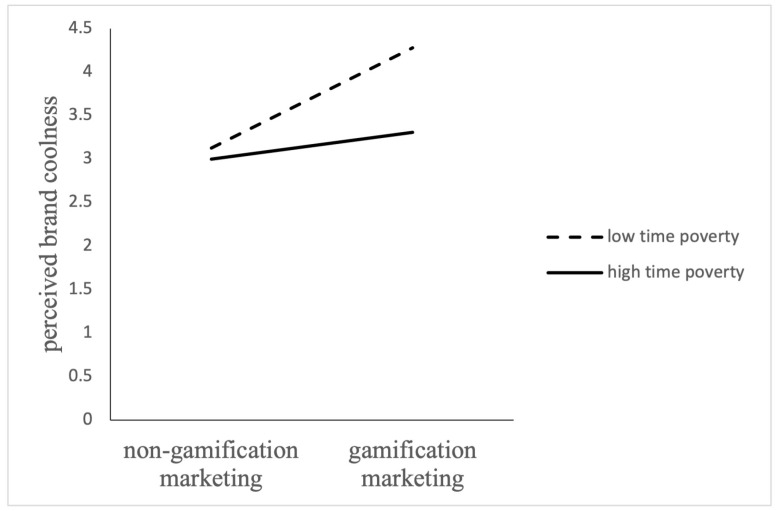
The Moderating Effect of Time Poverty on the Relationship Between Gamification Marketing and Perceived Brand Coolness.

The analysis of the moderating effect was conducted with perceived brand coolness as the dependent variable to verify the moderating effect of time poverty on brand coolness. As shown in Table 3 and Figure 5, for participants with low time poverty perception (M − 1SD) (*B* = 1.12, *t* = 1.89, *p* < 0.001), gamification had a significant positive effect on brand coolness. While for the participants with high time poverty perception (M + 1SD), gamification had a positive effect on perceived brand coolness, but the effect was less (B = 0.12, *t* = 1.38, *p* < 0.001).

The results show that the positive effect of gamification marketing on perceived brand coolness decreases with increasing perceived time poverty. So, Hypothesis 4 is supported.

## 5. Discussion

Brands are increasingly adopting gamification marketing activities to attract customer attention and interest. Drawing on the self-determination theory (SDT), our study provides converging evidence that gamified marketing significantly increases customer purchase intention through perceived brand coolness under the context of low time poverty.

Firstly, the study confirms the positive impact of gamification marketing on purchase intention. Consistent with prior research [6,85], gamification enhances user motivation, enjoyment, and engagement, particularly in challenging tasks or goal-oriented scenarios [27,86,87]. We found that an achievement-related mechanism of points and leaderboards with the marketing activity significantly affects purchase intention. This finding confirms the role of games with entertainment in attracting individuals, creating positive emotions and improving impressions of products [88]. Emotional experiences associated with gameplay can become a source of intrinsic motivation if they include pleasure, arousal, and dominance dimensions which would increase consumers’ absorption during gameplay [89]. This result is consistent with the self-determination theory (SDT) that people can be motivated by intrinsic motivations such as autonomy, competence, and relatedness [24,25,76]. A previous study showed that competence satisfaction as an intrinsic need can lead to autotelic behaviors [69], we can conclude that gamification marketing with points and leaderboards in our study would affect consumer behaviors. This result conforms with previous studies that a gaming system derived from a set of tools (i.e., points, levels, leaderboards) is the motivation for companies to provide autonomy and relatedness for customers.

Secondly, perceived brand coolness is identified as a crucial mediating factor in the relationship between gamification marketing and purchase intention which is a new research finding in the mediation mechanism. The result aligns with previous research indicating that gamification may not directly influence consumer behavior [5]. Instead, the impact was mediated by factors like perceived enjoyment and flow experience. As reported in the mediation test results, perceived brand coolness mediates the relationship between gamification marketing and purchase intention. The result agrees with the research discussed by Nobre and Ferreira [90] that gamification can be seen as an innovative branding tool to promote consumer interaction and participation in brand experiences. The obtained results from this study are consistent with the research studied by Xi and Hamari [91] that immersion-based gamification is positively associated with emotional brand engagement. Brand coolness requires that the game itself have a cool experience in order to connect the experience with the perception of the brand. The immersive experience, emotional relaxation, and achievement acquisition induced by gamification marketing will further enhance the positive perception of the target group towards the brand [92]. Our results extend the previous literature by identifying mediation effects of perceived brand coolness, responding to the calls for new and novel perspectives of gamification research [88].

Thirdly, time poverty acts as a moderator, influencing the relationship between gamification marketing and purchase intention. In addition, time poverty moderate and the relationship between gamification marketing and perceived brand coolness. Minimal attention has been paid to the role of time poverty in the gamified marketplace [49]. Our findings extend the boundary conditions research of gamification on the perspective of perceived brand coolness. High time poverty negatively impacts cognitive elaboration about the brand, reducing the effectiveness of gamification marketing. However, individuals with low time poverty are more likely to perceive brand coolness and show more purchase intention for gamification activities. Initial evidence found that when consumers experienced high time poverty to complete the game, their cognitive elaboration about the brand was diminished [20]. High time poverty causes attention occupation of individuals and leads them unable to allocate more attention resources to game marketing activities, reducing the cognitive function of brand value [18]. Individuals with high time poverty have less disposable time and are difficult to devote time and energy to their favorite activities, so they are prone to reduce irrelevant leisure activities [93]. Moreover, under the conditions of high time poverty, individuals will further magnify their negative emotions (such as irritability, and resistance), which may lead to a decrease in willingness to pay [94]. Generally, game activities that are set by competition mechanism would increase stress and emotional burden once individuals are in high time poverty [95]. Individuals with low time poverty, on the other hand, have a more leisurely pace of life and more relaxed emotion that are easier to concentrate [96].

In summary, this research demonstrates how gamification can enhance purchase intention from the perspective of perceived brand coolness, offering a novel contribution to the gamification literature. First, this study confirms that gamification positively influences purchase intention (H1), aligning with previous research. Prior studies have shown that gamification positively impacts consumer engagement, including satisfaction, purchase intention, and loyalty [41,42]. Additionally, Hypothesis 2, which posits that perceived brand coolness mediates the relationship between gamification marketing and purchase intention, is supported. This aspect has not been empirically explored in the literature of gamification and paying willingness. Our results provide a new explanation for the influence mechanism of gamification and purchase intention. Finally, Hypothesis 3 and 4 are confirmed, indicating that time poverty moderates the relationship between gamification and purchase intention as well as between gamification and perceived brand coolness. While time pressure has been shown to negatively moderate the relationship between gamified interactions and self-brand connections through cognitive brand engagement [20], there is no existing literature addressing whether time poverty moderates the relationship between gamification and purchase intention as well as between gamification and perceived brand coolness. Our study introduces Hypothesis 3 and 4 as new perspectives to the exploration of boundary conditions in gamification research.

## 6. Conclusions

### 6.1. Theoretical Contributions

Firstly, the study expands the study of gamification, shedding light on the nuanced relationship between gamification and purchase intention. It significantly contributes to the existing gamification studies by examining both the mediating effect of perceived brand coolness and the moderating effect of time poverty. The mediating effect of perceived brand coolness discussed in this study broadens gamification marketing research for the relationships that were discussed less in previous empirical studies. Secondly, the study provides a new perspective on how gamified marketing works by taking perceived brand coolness as a mediating variable. It is a supplement to the existing research that takes flow experience or perceived funny as the mediating variable. Perceived brand coolness provides a new explanation for the effect of gamification on consumer behavior. Finally, introducing time poverty to the model offers conclusive evidence for understanding the boundary conditions of gamification marketing. Our results indicate that whether the gamification leads to a positive outcome depends on consumers’ possessed time. Customers who are in low time poverty condition perceive more brand coolness and are more likely to purchase the brand product. The boundary condition discussed in our study is also a supplement to the study of conditions of the self-determination theory (SDT).

### 6.2. Practical Implications

Firstly, this research has implications for firms that use tools to attract users to pay for a brand product. Gamification activities can be simultaneously used by marketers as they provide users perception of brand coolness. Customers playing the games with interactions and challenges were more engaged and felt stronger connections with the brand, thus causing more pay willingness. Marketing managers and game designers can integrate gamified elements with unique design techniques into non-game contexts. For example, participants can earn rewards based on leaderboard rankings on shopping websites by inviting friends to play games embedded in these platforms. Additionally, strategies such as using cosplay (e.g., farmer, driver, gardener) can motivate more consumers to engage in brand-related activities. Secondly, gamification activities that were set by identifying brand coolness as a mediator were efficient to purchase intention. Firms can design games with cool elements (i.e., fashion, artificial intelligence, subcultural form) [97]. Brand managers could also implement gamification strategies that emphasize autonomy and novelty to enhance the perceived coolness of a brand. Building on this understanding, marketing managers and game designers could incorporate virtual idols (e.g., Hatsune Miku, Kagamine Rin/Len, Luo Tianyi) with cool images into gamification activities. By merging interactive challenges, shared achievements, and opportunities for discussions, these activities can further enhance the coolness of the brand [98]. Thirdly, game designers providing users with enough time to engage in gamification marketing activities were well appreciated by customers. The results showed that not all gamified interactions would obtain good results. When consumers are in high time poverty during their participation in gameplay, consumers may feel controlled and under high pressure. The effect of gamification on perceived brand coolness and purchase intention would decrease compared to low time poverty conditions. Game designers are advised to create games that allow customers to complete them at their own pace, with the option to exit and return without losing earned points. Furthermore, firms can identify target consumers who are experiencing low time poverty by analyzing online data. Providing a relaxing atmosphere (e.g., light music, humorous storylines) can help make consumers feel more at ease and improve their overall experience [99]. In conclusion, this study provided valuable insights into the relationships between gamification marketing, perceived brand coolness, and time poverty, offering practical implications for marketers seeking to enhance consumer engagement and purchase intention.

### 6.3. Limitations and Future Directions for Research

This research does not delve into specific game elements or game sessions and how those affect purchase intentions. Future research could explore these details, investigating the impact of game design elements (i.e., task settings, distribution timing, challenges) on behavioral intentions. In addition, the study acknowledges a possible positive bias in the data due to using the young mostly as participants. Future research should consider inviting diverse participants to obtain a broader perspective, especially from those who are not familiar with game playing. Nevertheless, the result of our study shows that the high age group (27–45) and low age group (19–24) in our study samples are balanced in the gamification effect on purchase intention. The gender ratio is consistent with real life in lipstick consuming market. Samples in our study are representative to some contexts. Lipstick as a stimulus in our study is a limitation because only one certain product type is involved. Future research should pay more attention to other product types.

In conclusion, this study not only advances theoretical understanding by exploring new variables and their effects on consumer behavior but also provides actionable insights for marketers and gamification designers to enhance customer brand engagement and drive purchase intention. The emphasis on the interaction between gamification, perceived brand coolness, and time poverty contributes to a more comprehensive understanding of the dynamics of consumer decisions.

## Figures and Tables

**Table 1:** Demographic Description of Participants (N = 184).

Characteristics	Item	Frequency	Percentage
Gender	Men	39	21.20%
	Women	145	78.80%
Age	1~18	12	6.52%
	19~30	159	86.41%
	31~45	6	3.26%
	46~60	7	3.80%
Monthly income	0~1000	93	50.54%
	1001~2000	43	23.37%
	2001~5000	28	15.22%
	Above 5000	20	10.87%

**Table 2:** Testing the Mediating Model of Perceived Brand Coolness.

	Purchase Intension			Perceived Brand Coolness			Purchase Intension		
variable	B	t	95% CI [LI, UI]	B	t	95% CI [LI, UI]	B	t	95% CI [LI, UI]
(constant)	3.43 ***	26.19	[3.17, 3.69]	3.49 ***	27.97	[3.24, 3.74]	1.15 *	5.92	[0.77, 1.53]
Gamification marketing	0.64 ***	3.43	[0.27, 1.01]	0.67 ***	3.78	[0.32, 1.02]	0.24	1.83	[−0.19, 0.49]
Perceived brand coolness							0.68 ***	13.66	[0.58, 0.78]
R^2^	0.06			0.07			0.55		
F (df)	11.78			14.26			107.74		

Note. *** means *p* < 0.001; * means *p* < 0.05. CI = confidence interval of the difference; LI = lower interval; UI = upper interval.

**Table 3:** Testing the Moderating Model of Time Poverty.

	Perceived Brand Coolness			Purchase Intension		
Variable	B	t	95% CI [LI, UI]	B	t	95% CI [LI, UI]
(constant)	3.70 ***	40.57	[3.52, 3.88]	1.04 ***	4.86	[0.62, 1.46]
Gamification marketing	0.58 ***	3.17	[0.22, 0.94]	0.12 ***	1.89	[0.15, 0.40]
Time poverty	−0.12 ***	−2.05	[−0.23, −0.01]	−0.13	−0.63	[−0.12, 0.06]
Perceived brand coolness				0.74 ***	13.73	[0.63, 0.84]
Gamification marketing × time poverty	−0.35 ***	−3.12	[−0.58, −0.13]	−0.21 *	−1.55	[−0.37, −0.04]
R^2^	0.14			0.08		
F (df)	9.48			15.24		

Note. *** means *p* < 0.001; * means *p* < 0.05. CI = confidence interval of the difference; LI = lower interval; UI = upper interval.

## Data Availability

The data that support the findings of this study are available from the corresponding author upon reasonable request.
